# Recent advances in the study of chloroplast gene expression and its evolution

**DOI:** 10.3389/fpls.2014.00061

**Published:** 2014-02-25

**Authors:** Yusuke Yagi, Takashi Shiina

**Affiliations:** ^1^Faculty of Agriculture, Kyushu UniversityFukuoka, Japan; ^2^Graduate School of Life and Environmental Sciences, Kyoto Prefectural UniversityKyoto, Japan

**Keywords:** chloroplast, transcription, PEP, NEP, pTAC, nucleoid

## Abstract

Chloroplasts are semiautonomous organelles which possess their own genome and gene expression system. However, extant chloroplasts contain only limited coding information, and are dependent on a large number of nucleus-encoded proteins. During plant evolution, chloroplasts have lost most of the prokaryotic DNA-binding proteins and transcription regulators that were present in the original endosymbiont. Thus, chloroplasts have a unique hybrid transcription system composed of the remaining prokaryotic components, such as a prokaryotic RNA polymerase as well as nucleus-encoded eukaryotic components. Recent proteomic and transcriptomic analyses have provided insights into chloroplast transcription systems and their evolution. Here, we review chloroplast-specific transcription systems, focusing on the multiple RNA polymerases, eukaryotic transcription regulators in chloroplasts, chloroplast promoters, and the dynamics of chloroplast nucleoids.

## INTRODUCTION

Chloroplasts are believed to have arisen from an endosymbiotic event between a photosynthetic cyanobacterium and the ancestral eukaryotic cell. Although chloroplasts of modern plants and algae have retained the genome of the symbiont, that genome has markedly shrunk over endosymbiotic evolution. Many chloroplast-encoded genes were lost or transferred to the nucleus soon after endosymbiosis. Thus, chloroplast genomes of extant land plants have only 50 protein-coding genes involved in photosynthesis, gene expression, lipid metabolism and other processes, 30 tRNA genes and full sets of rRNA genes. In spite of their small genomes (0.15 Mbp in land plant chloroplasts versus 3 Mbp in cyanobacteria), chloroplast gene expression is regulated by more complex systems compared to the simple prokaryotic regulatory system. Chloroplast gene expression is mediated by two distinct types of RNA polymerase (RNAP) and is highly dependent on post-transcriptional regulation, such as the processing of polycistronic transcripts, intron splicing and RNA editing. Moreover, recent RNA-seq analyses of chloroplast transcripts identified unexpected diversifications of RNA molecules, such as non-coding and antisense RNAs (Hotto et al., 2011; Zhelyazkova et al., 2012). However, the genes encoded in chloroplast genomes are insufficient to regulate their complicated gene expression, and so the chloroplast gene expression machinery includes various nucleus-encoded regulatory components.

Although basic chloroplast gene expression is mediated by prokaryotic machineries derived from the ancestral cyanobacterium, chloroplasts lost their homologs of bacterial regulatory elements such as transcription factors (TFs) and nucleoid proteins at an early stage of their evolution. Genomics and proteomics analyses of chloroplast proteins in *Arabidopsis thaliana* have suggested that 60% of the chloroplast proteome may have been newly acquired from the nuclear genome of host cells after the endosymbiotic event (Abdallah et al., 2000). Indeed, recent analyses of the chloroplast nucleoid proteins identified many non-bacterial components that play critical roles in chloroplast gene expression including transcription, post-transcriptional RNA processing, and translation. Here, we summarize the current knowledge regarding the chloroplast gene expression system.

## TWO BASIC CHLOROPLAST TRANSCRIPTION MACHINERIES WITH DIFFERENT EVOLUTIONARY ORIGIN

**FIGURE 1:**
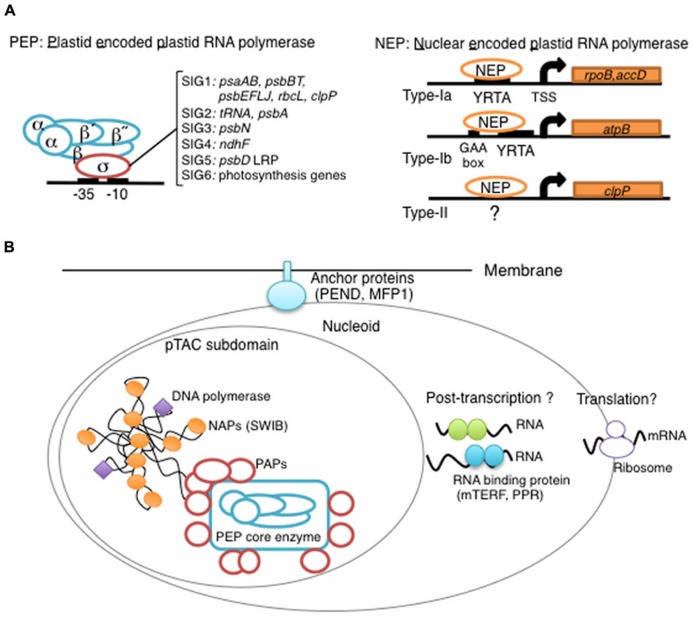
**Overview of chloroplast transcription.** **(A)** Basic transcriptional machinery in higher plants. Higher plants have two distinct types of chloroplast RNA polymerase: plastid-encoded plastid RNA polymerase (PEP; left panel) and nucleus-encoded plastid RNA polymerase (NEP; right panel). PEP is a bacterial-type multi-subunit RNA polymerase composed of the core enzymatic subunits α, β, β′, β″ (blue) and a sigma subunit (red) that is responsible for promoter recognition. Plastid sigma factors are divided into six subgroups, SIG1–SIG6, and selectively recognize bacterial-type promoters in the plastid. NEP (right panel) is a monomeric enzyme that resembles mitochondrial T7-type RNA polymerases. NEP is involved in the transcription of housekeeping genes such as *rpo* genes for PEP core subunits, and ribosomal protein-coding genes. Positioned upstream of genes transcribed by NEP are three distinct types of promoter structures (Type-Ia, Type-Ib, and Type-II). **(B)** The chloroplast nucleoid subdomain and its components. Chloroplast nucleoids are attached to the membrane (envelope or thylakoid) by anchor proteins (PEND and MFP1). The plastid transcription active chromosome (pTAC) is one of the nucleoid subdomains, which contains the transcription factory. Chloroplast genomic DNA is packed by chloroplast-specific nucleoid-associated proteins (NAPs; orange circle). The mature chloroplast contains a large PEP complex with several PEP associate proteins (PAPs; red circles). Recent proteome analysis suggested that chloroplast nucleoids contain additional subdomains, which regulate post-transcriptional RNA maturation and translation.

Chloroplast gene expression is largely dependent on prokaryotic machineries derived from the ancestral cyanobacterium. The bacterial multi-subunit RNAP is composed of a core Rpo complex, which has the catalytic enzyme activity, and a sigma factor, which recognizes promoter sequences (Ishihama, 2000). Chloroplasts contain the bacterial-type RNAP, called plastid-encoded plastid RNAP (PEP), which shares functional similarity with the bacterial RNAP (Igloi and Kossel, 1992; **Figure 1A**) However, all genes for chloroplast sigma factors have been transferred to the nuclear genome, whereas genes for core subunits are typically retained in the chloroplast genome as *rpoA*, *rpoB, rpoC1*, and *rpoC2*.

Early work demonstrated that almost all photosynthesis-related transcripts are significantly reduced in PEP-deficient plants, such as ribosome-deficient mutants of barley (*Hordeum vulgare*), *iojap* mutants of maize and tobacco mutants with disrupted *rpo* genes generated by gene targeting using chloroplast transformation (Han et al., 1992; Hess et al., 1993, 1994; Allison et al., 1996; De Santis-MacIossek et al., 1999), whereas a set of housekeeping genes are still active in these mutants. The inhibitor sensitivity of this transcription activity is similar to that of phage T7 RNAP, but not to that of bacterial RNAP (Kapoor et al., 1997; Sakai et al., 1998). In *Arabidopsis*, three phage-type RNAP genes were identified and their subcellular localization was determined (*RpoTp*:* chloroplasts*, *RpoTm*:* mitochondria*, *RpoTmp*:* chloroplast and mitochondria*; Hedtke et al., 1997, 2000; Weihe and Borner, 1999; **Figure 1A**). RpoTmp and RpoTp likely represent nuclear encoded RNAP (NEP) enzyme in chloroplasts [reviewed in (Liere et al., 2011)], while RpoTmp has been identified in dicotyledonous plants such as *Arabidopsis* and tobacco but not in monocotyledonous plant genomes (Chang et al., 1999; Ikeda and Gray, 1999; Emanuel et al., 2004).

Only one *RpoT* gene has been identified in green algae, such as *Chlamydomonas reinhardtii*, *Ostreococcus tauri*, and *Thalassiosira pseudonana*, which likely encodes mitochondrial RNAP (Maier et al., 2008). Similarly, the genome of the lycophyte *Selaginella moellendorffii* contains only one *RpoT* gene, the product of which has been shown to target mitochondria (Yin et al., 2009). On the other hand, the moss *Physcomitrella patens* has three *RpoT* genes. However, all GFP-fused moss RpoTs were detected exclusively in mitochondria, suggesting that the moss *RpoT* genes also encode mitochondrial RNAP (Kabeya et al., 2002; Richter et al., 2002, 2013). Moreover, phylogenetic analysis of plant *RpoT* genes suggests that NEP appeared through the gene duplication of mitochondrial RNAP after the separation of angiosperms from gymnosperms (Yin et al., 2010).

## SELECTIVE CHLOROPLAST TRANSCRIPTION BY PEP AND NEP

Chloroplast genes can be categorized into three subgroups, classes I–III: class I photosynthesis-related genes are mainly transcribed by PEP; Class II includes many housekeeping genes (*clpP* and the *rrn* operon) that are transcribed by both PEP and NEP; class III genes (*accD* and the *rpoB* operon) are exclusively transcribed by NEP (Allison et al., 1996; Hajdukiewicz et al., 1997).

PEP recognizes standard chloroplast promoters resembling the bacterial σ^70^ type promoters with -10 and -35 consensus elements (Gatenby et al., 1981; Gruissem and Zurawski, 1985; Strittmatter et al., 1985; Shiina et al., 2005; **Figure 1A**). A genome-wide mapping of transcription start sites (TSSs) by RNA sequencing in barley green chloroplasts demonstrated that 89% of the mapped TSSs have a conserved -10 element (TAtaaT) at three to nine nucleotides upstream, while the -35 element was mapped upstream of the -10 element in only 70% of the TSSs (Zhelyazkova et al., 2012). These results suggest that most genes are transcribed from σ^70^-type promoters by PEP in green leaves.

Higher plants have multiple sigma factors that are expected to confer promoter specificity upon the PEP core complex (Shiina et al., 2005; Lerbs-Mache, 2011). Molecular genetic analyses revealed that SIG2 is responsible for the transcription of a group of tRNA genes, but not photosynthesis genes (Kanamaru et al., 2001), while SIG6 is essential for the transcription of a wide range of photosynthesis-related genes at an early stage of chloroplast development (Ishizaki et al., 2005). It seems likely that SIG2 and SIG6 work in cooperation during light-dependent chloroplast development (Hanaoka et al., 2003; Ishizaki et al., 2005). In addition, SIG3 and SIG4 have been shown to specifically target *psbN* and *ndhF* genes in *Arabidopsis* (Favory et al., 2005; Zghidi et al., 2007). Recently, ChIP analysis of SIG1 revealed the target genes (*psaAB*, *psbBT*, *psbEFLJ*, *rbcL*, and *clpP*; Hanaoka et al., 2012). SIG5 is a unique sigma factor whose expression is rapidly induced by various environmental stresses such as a high osmolarity, or salinity, a low temperature as well as high-light stress (Tsunoyama et al., 2002; Nagashima et al., 2004). SIG5 likely recognizes specific promoters, including the *psbD* light-responsive promoter (LRP), and mediates stress-induced transcription in chloroplasts (Nagashima et al., 2004; Tsunoyama et al., 2004). Taken together, it is likely that each chloroplasts sigma factor is responsible for the transcription of a distinct set of genes, and plays specific roles in transcriptional regulation in response to developmental and/or environmental cues.

Phylogenetic analysis revealed that chloroplast sigma factors are related to essential group 1 and non-essential group 2 sigma factors in bacteria. *Chlamydomonas*, a single-celled green alga, possesses a single sigma factor that is related to SIG2 in land plants, suggesting the absence of multiple sigma factor-mediated transcriptional regulation in chloroplasts. Endosymbiosis of ancestral cyanobacteria in plant cells may have reduced the need for transcriptional regulation in chloroplasts and caused the reduction of the number of sigma factors in green algae. On the other hand, in liverwort (*M. polymorpha *L*.*) and moss (*P. patens*), three sigma factors related to SIG1, SIG2, and SIG5 are encoded in the nucleus (Shiina et al., 2009; Ueda et al., 2013). The multiple sigma factors in bryophytes may show a promoter preference and play roles in tissue-specific and stress-responsive transcriptional regulation in chloroplasts (Hara et al., 2001; Ichikawa et al., 2004; Kanazawa et al., 2013; Ueda et al., 2013).

Most NEP promoters (*rpoB*, *rpoA*, and *accD*) share a core sequence, the YRTA motif (type-Ia; Liere and Maliga, 1999; Weihe and Borner, 1999; Hirata et al., 2004; **Figure 1A**). The YRTA motif is similar to motifs found in promoters of plant mitochondria (Binder and Brennicke, 2003; Kuhn et al., 2005). In addition, GAA-box has been identified upstream of the YRTA motif in a subclass of NEP promoters (type-Ib; Kapoor and Sugiura, 1999). In contrast to these standard NEP promoters, type-II NEP promoters mapped upstream of the dicot *clpP* gene lack the YRTA motif and are dependent on downstream sequences of the TSS (Weihe and Borner, 1999). Furthermore, it has been shown that the *rrn* operon and certain tRNAs are transcribed from other non-consensus-type NEP promoters [Reviewed by (Liere et al., 2011)].

Although the class I genes have been clarified as being exclusively transcribed by PEP, the genome-wide mapping of TSSs in barley revealed that most genes including photosynthesis genes have both PEP and NEP promoters. It seems likely that NEP supports transcription of photosynthesis genes at the early stage of seedling greening (Zhelyazkova et al., 2012). Interestingly, 73% of NEP-dependent TSSs possess the YRTA motif typical for type-Ia and -Ib NEP promoters, whereas GAA-boxes have been barely mapped upstream of the barley NEP promoters. These results suggest that type-Ia, but not type-Ib NEP promoters play a major role in transcription by NEP in barley chloroplasts. In contrast, type-II NEP promoters, which are dependent on downstream sequences of the TSSs, were identified in barley as well as tobacco.

## THE LARGE TRANSCRIPTION COMPLEX IN HIGHER PLANT CHLOROPLASTS

Two types of PEP-containing preparation have been biochemically isolated in mustard and *Arabidopsis*: soluble RNAP (sRNAP) and plastid transcriptionally active chromosome (pTAC) attached to chloroplast membranes (Hess and Borner, 1999). Transcription by sRNAP is dependent on exogenously added template DNA, whereas the pTAC can initiate transcription from the endogenous chloroplast DNA (Igloi and Kossel, 1992; Krause et al., 2000). Interestingly, protein compositions of highly purified sRNAP fractions are dependent on chloroplast development (Pfannschmidt and Link, 1994). The sRNAP of etioplasts in dark-grown leaves is a naked RNAP without additional subunits similar to the *E. coli* RNAP core complex. Etioplasts convert to photosynthetically active chloroplasts in the presence of light. During chloroplast development in mustard, the RNAP develops a more complex form that contains 13 additional polypeptides (Pfannschmidt and Link, 1994). It seems likely that the simple sRNAP in etioplasts converts to a more complex sRNAP in chloroplasts by recruiting additional components during chloroplast development.

Proteomic analyses of pTAC fractions isolated from mature chloroplasts of *Arabidopsis* and mustard have identified 35 polypeptides including 18 novel proteins termed pTAC1–pTAC18, in addition to PEP core subunits, DNA polymerase, DNA gyrase, Fe-dependent superoxide dismutases (FeSODs), phosphofructokinase–B type enzymes (PFKB1 and PFKB2), thioredoxin, and three ribosomal proteins (Pfalz et al., 2006). DNA- and/or RNA-binding domains, protein–protein interaction domains, or epitopes with other reported cellular functions have been identified in some of pTAC proteins. Most *Arabidopsis* knockout mutants of pTAC proteins exhibit seedling-lethal symptoms or chlorophyll-deficient phenotypes. PEP-dependent transcription is significantly impaired in the pTAC mutants, whereas NEP-dependent transcription is up-regulated. These phenotypes and chloroplast gene expression patterns are reminiscent of those of *rpo *mutants (Allison et al., 1996; Hajdukiewicz et al., 1997), suggesting a critical role for pTAC proteins in PEP transcription.

Affinity purification of the tobacco PEP (Suzuki et al., 2004) and more recent analysis of subunits of the PEP complex in mustard (Steiner et al., 2011) and tobacco complex identified at least 10 PEP-associated proteins (PAPs). Recently, chromatin immunoprecipitation assays were performed with one of the typical PAPs, pTAC3/PAP1. The results revealed that pTAC3/PAP1 associates with the PEP complex in all three steps of the transcription cycle including initiation, elongation and termination, suggesting that pTAC3/PAP1 is an essential component of the chloroplast PEP complex (Yagi et al., 2012). Several studies on protein–protein interactions among PAPs have been reported [reviewed in (Pfalz and Pfannschmidt, 2013)]. Almost all PAP genes, except for *Trx-z*, are conserved among all land plants, but not in the green alga *Chlamydomonas*. It seems likely that terrestrial plants may have acquired non-cyanobacterial novel PEP components during land plant evolution to regulate plastid transcription (Pfalz and Pfannschmidt, 2013).

It has been suggested that a series of checkpoints control the establishment of the chloroplast transcription machinery (Steiner et al., 2011; Pfalz and Pfannschmidt, 2013). In imbibed seeds, predominant NEP is responsible for transcription of housekeeping genes. NEP also transcribes chloroplast-encoded *rpo* genes for PEP core subunits to produce a basic PEP-B complex (NEP–PEP cascade). PEP-B is responsible for the major activity in etioplasts and in an early stage of greening. This step may be the first checkpoint. Subsequently, PEP-B associates with PAPs and converts them into a larger PEP complex (PEP-A) during light-dependent chloroplast development. PEP-A formation is strictly dependent on light. Indeed, it has been reported that expression of the *pTAC3*/*PAP1* gene is induced by light during the greening process (Yagi et al., 2012). PAP mutants mostly show the aberrant development of chloroplasts and transcription of chloroplast-encoded genes, suggesting their essential roles in PEP-A. Furthermore, recent genome-wide analysis of the chloroplast transcriptome revealed reduced expressions of numerous chloroplast tRNAs in several PAPs mutants (pTAC2, pTAC12, MurE, PRIN2), suggesting that PAPs play a major role in tRNA transcription in chloroplasts (Williams-Carrier et al., 2014). Thus PAPs are also responsible for protein translation in chloroplasts. Therefore, the assembly of PAPs in the PEP-A complex may be the second checkpoint in the establishment of the chloroplast transcription machinery. To prevent uncontrolled chloroplast development under adverse conditions, these check points likely play critical roles in the control of chloroplast gene transcription.

## THE PLASTID NUCLEOIDS: DYNAMICS AND UNIQUE COMPONENTS

The plastid DNA exists as large protein-DNA complexes named the plastid nucleoid. Plastid nucleoids contain an average of 10–20 copies of the plastid DNA (Kuroiwa, 1991), and their size, shape, and distribution vary depending on the plastid type (Miyamura et al., 1986; Sato et al., 1997). Each chloroplast contains ~20 nucleoids that are randomly located on the thylakoid membranes. Immature proplastids in seeds contain only one nucleoid that is located at the center of the organelle. The plastid nucleoids divide into a few small dots and redistribute to the inner envelope membranes during early chloroplast development. At a later stage of chloroplast development, nucleoids are relocated to the thylakoid membranes. It has been suggested that plastid nucleoid organization and dynamics are involved in the regulation of plastid function, gene expression and differentiation. Two DNA-binding proteins, PEND and MFP1, are likely responsible for the association of nucleoids with chloroplast membranes (Sato et al., 1998; Jeong et al., 2003; **Figure 1B**).

**FIGURE 2:**
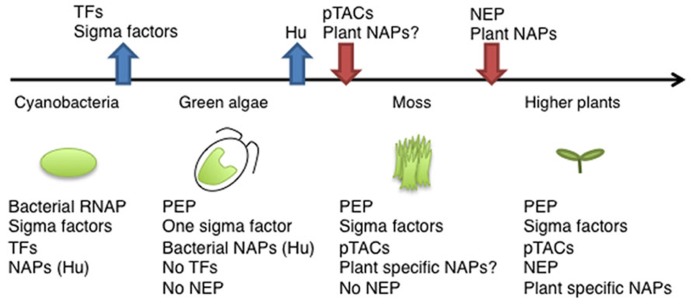
**Evolution of components of the chloroplast transcription machinery.** Ancient cyanobacteria have a prototype of PEP including Rpo subunits and several types of sigma factors, several transcription factors (TFs), and various nucleoid-associated proteins (NAPs). After endosymbiosis, the primary chloroplast lost its sigma factors, except the σ^70^ type, and all TFs. During the evolution of land plants, chloroplasts acquired more complicated transcription machinery with a variety of pTACs. In higher plants, there are multiple RNA polymerases (PEP, multiple sigma factors, PEP-pTAC complex, and NEP), and plant-specific NAPs.

In *E. coli*, chromosome DNA packaging patterns affect gene expression, and are regulated by nucleoid-associated proteins (NAPs) such as HU, H-NS, and FIS [reviewed by (Dillon and Dorman, 2010)]. Among bacterial NAPs, HU is one of the major DNA-binding proteins and is involved in chromosome DNA packaging. HU-like proteins (HLPs) are conserved in cyanobacteria, the red alga *Cyanidioschyzon merolae* (Kobayashi et al., 2002), and the green alga *Chlamydomonas* (Karcher et al., 2009). The HLP in *Chlamydomonas* has roles in nucleoid maintenance and gene expression, indicating conserved roles of HU during chloroplast evolution (Karcher et al., 2009). However, land plants including mosses and flowering plants have not only lost the HU genes, but also other prokaryotic DNA-binding proteins (**Figure 2**). Nevertheless, atomic force microscopy observations revealed that plastid nucleoids are highly organized and form a beads-on-a-string structure similar to that observed in bacterial nucleoids, suggesting that another host cell-derived DNA-binding protein took over the functions of HU (Melonek et al., 2012). Recently, eukaryotic SWIB (SWI/SNF complex B) domain containing proteins have been identified from the proteome of a further-enriched pTAC fraction (TAC-II) of spinach chloroplasts (Melonek et al., 2012). SWIB4 that has a histone H1 motif, can functionally complement an *E. coli *mutant lacking the histone-like nucleoid structuring protein H-NS, indicating that SWIB4 is the most likely counterpart of the bacterial NAPs in chloroplasts. EM observation of isolated pTAC identified chromatin-like beaded structures with several protruding DNA loops, suggesting that pTACs represent a subdomain of the chloroplast nucleoid (Yoshida et al., 1978; Briat et al., 1982). These findings suggest that pTAC forms a central core of the plastid nucleoid and a transcription factory (**Figure 1B**).

The proteomes of highly enriched nucleoid fractions have been characterized in maize proplastids and mature chloroplasts (Majeran et al., 2012). As expected, the chloroplast nucleoids contain all PEP core Rpos and PAPs, and almost all other pTAC proteins. Furthermore, additional proteins involved in post-transcriptional processes, such as pentatricopeptide repeat proteins (PPR proteins), mitochondrial transcription factor (mTERF)-domain proteins, 70S ribosomes and ribosome assembly factors have been identified in the proteome of the chloroplast nucleoids, suggesting that several post-transcriptional events including RNA processing, splicing and editing, and translation, occur in nucleoids, and that these processes are co-regulated with transcription (**Figure 1B**). Human mitochondrial nucleoids have been shown to form layered structures, the central core involved in replication and transcription, and the peripheral region where translation and complex assembly may occur (Bogenhagen et al., 2008). By analogy, the further characterization of plastid nucleoids will provide insights into the structural specialization of plastid nucleoids; DNA maintenance and transcription in a core domain and various aspects of RNA metabolism in several subdomains.

## PERSPECTIVE

Recent proteomic and transcriptomic researches and the development of novel ChIP and imaging technologies have advanced the understanding of the molecular basis of RNAP complexes and nucleoid architecture. In land plants, neither the nuclear nor chloroplast genome encodes prokaryotic transcription factors and nucleoid proteins, whereas chloroplasts retain prokaryotic-type RNAP (**Figure 2**). In fact, land plants have a number of novel host cell-derived transcription regulators and DNA-binding proteins that are involved in the regulation of chloroplast transcription. Thus, it seems likely that chloroplast transcription is mediated by a hybrid system of prokaryotic and eukaryotic origin. Further molecular characterization of pTACs and plastid nucleoid proteins would provide novel insights into the unique plastid gene expression system and as yet known mechanisms of plastid differentiation.

## Conflict of Interest Statement

The authors declare that the research was conducted in the absence of any commercial or financial relationships that could be construed as a potential conflict of interest.
